# A prognostic model for predicting progression-free survival in patients with advanced non-small cell lung cancer after image-guided microwave ablation plus chemotherapy

**DOI:** 10.1007/s00330-023-09804-9

**Published:** 2023-06-15

**Authors:** Fanhao Kong, Honglan Yang, Qiaoxia Wang, Zhigang Wei, Xin Ye

**Affiliations:** 1 The First Clinical Department, China Medical University, 155 Nanjingbei Road, Liaoning Province Shenyang, China; 2 Department of Oncology, Dongying People’s Hospital, 317 Nanyi Road, Dongying, Shandong Province China; 3 Department of Respiratory, Dongying People’s Hospital, 317 Nanyi Road, Shandong Province Dongying, China; 4 Department of Oncology, The First Affiliated Hospital of Shandong First Medical University & Shandong Provincial Qianfoshan Hospital, Shandong Lung Cancer Institute, Shandong Key Laboratory of Rheumatic Disease and Translational Medicine, Jinan, Shandong Province China; 5 Cheeloo College of Medicine, Shandong University, Jinan, Shandong Province China

**Keywords:** Carcinoma, Non-small-cell lung, Microwave, Lung, Prognosis

## Abstract

**Objectives:**

This study aimed to build and validate a prediction model that can predict progression-free survival (PFS) in patients with advanced non-small cell lung cancer (NSCLC) after image-guided microwave ablation (MWA) plus chemotherapy.

**Methods:**

Data from a previous multi-center randomized controlled trial (RCT) was used and assigned to either the training data set or the external validation data set according to the location of the centers. Potential prognostic factors were identified by multivariable analysis in the training data set and used to construct a nomogram. After bootstraps internal and external validation, the predictive performance was evaluated by concordance index (C-index), Brier Score, and calibration curves. Risk group stratification was conducted using the score calculated by the nomogram. Then a simplified scoring system was built to make risk group stratification more convenient.

**Results:**

In total, 148 patients (training data set: *n* = 112; external validation data set: *n* = 36) were enrolled for analysis. Six potential predictors were identified and entered into the nomogram, including weight loss, histology, clinical TNM stage, clinical N category, tumor location, and tumor size. The C-indexes were 0.77 (95% CI, 0.65–0.88, internal validation) and 0.64 (95% CI, 0.43–0.85, external validation). The survival curves of different risk groups also displayed significant distinction (*p* < 0.0001).

**Conclusions:**

We found weight loss, histology, clinical TNM stage, clinical N category, tumor location, and tumor size were prognostic factors of progression after receiving MWA plus chemotherapy and constructed a prediction model that can predict PFS.

**Clinical relevance statement:**

The nomogram and scoring system will assist physicians to predict the individualized PFS of their patients and decide whether to perform or terminate MWA and chemotherapy according to the expected benefits.

**Key Points:**

*• Build and validate a prognostic model using the data from a previous randomized controlled trial to predict progression-free survival after receiving MWA plus chemotherapy.*

*• Weight loss, histology, clinical TNM stage, clinical N category, tumor location, and tumor size were prognostic factors.*

*• The nomogram and scoring system published by the prediction model can be used to assist physicians to make clinical decisions.*

**Supplementary Information:**

The online version contains supplementary material available at 10.1007/s00330-023-09804-9.

## Introduction

According to GLOBOCAN 2020, lung cancer, the second most common cancer for both sexes and the most common for men, ranked first in the cause of cancer mortality in 2020 [1]. Accounting for 85%, non-small cell lung cancer (NSCLC) is the primary type of lung cancer [2]. Surgical resection with curative intent is the recommended treatment for early-stage (stage I, stage II, and stage IIIA) NSCLC [3]. However, more than two-thirds of NSCLC patients are diagnosed at the advanced stage and most are not proper candidates for surgery [4].

Thermal ablation has recently been considered to be an optional therapy for advanced NSCLC. Microwave ablation (MWA), radiofrequency ablation (RFA), and cryoablation are the three most commonly used ablation categories, in which MWA has lots of advantages over the other techniques, such as less “heat‑sink” effect and better convection [5]. But we still cannot neglect the discrepant recurrence rate of MWA, which ranges from 9 to 37% and may lead to a bad prognosis for patients receiving MWA [6]. To optimize this circumstance, previous studies have found that combined MWA and chemotherapy can prolong the progression-free survival of NSCLC patients [7–9].

At present, there is no prediction model focusing on the prognosis of patients with advanced NSCLC that received MWA and chemotherapy. Two former prediction models, which targeted predicting the local progression-free survival and overall survival (OS) of NSCLC patients treated with MWA, failed to limit the stage of NSCLC and clarify whether to combine chemotherapy with MWA [10, 11]. In order to determine the proper circumstance of utilizing MWA plus chemotherapy for patients with advanced NSCLC, a prognostic prediction model constructed with high-quality data is urgently needed.

In this study, we built and validated a prediction model to predict the 1-year PFS of patients with advanced NSCLC who received MWA and chemotherapy and published a nomogram and a simplified scoring system for clinical application.

## Materials and methods

### Data source and patient population

The data utilized to construct and validate this prediction model was extracted from a former multi-institutional RCT (NCT02455843) which was performed at 14 sites in China [12]. Treatment was carried out from March 1, 2015, to June 20, 2017, and the last follow-up time was October 26, 2017. Ethical approval was obtained by institutional review boards from all participating centers.

To enroll the appropriate candidates, patients’ inclusion and exclusion criteria were formulated by the researchers (Table 1). All the selected candidates were confirmed to have NSCLC at the clinical stage of IIIB/IV or recurrence after radical surgery, who were then randomly divided into chemotherapy group and MWA plus chemotherapy group by a centrally controlled system, which was checked by a trial-independent statistician. A form was made to collect all corresponding data, including sociodemographic data, MWA-related data, chemotherapy information, presence of any ablation-related complications, and endpoint information, which can be found in Table 2. And we utilized the data from the MWA plus chemotherapy group was employed to build the prediction model.

**Table 1:** Inclusion and exclusion criteria

Inclusion criteria
1. Age ≥ 18 years
2. Pathologically verified NSCLC at clinical stage of IIIB/IV or recurrence after radical surgery
3. ECOG PS 0–2
4. ≥ 1 measurable tumor site besides the ablative sites
5. Restricted peripheral lung cancer with normal hepatic, renal, and bone marrow functions
6. EGFR mutations/EML4-ALK fusion genes unknown/negative or mutant but targeted therapy refused by patient
7. Life expectancy > 3 months
Exclusion criteria
1. History of primary tumor other than lung cancer
2. Uncontrolled symptomatic brain metastases
3. Severe interstitial lung diseases
4. Acute myocardial infarction occurred 6 months before randomization
5. Platelet count < 100,000/μL

The 7th edition of the American Joint Committee on Cancer TNM staging system was used. *NSCLC*, non–small cell lung cancer; *ECOG PS*, Eastern Cooperation Oncology Group Performance Status; *EGFR*, epidermal growth factor receptor; *EML4-ALK*, echinoderm microtubule-associated protein-like 4-anaplastic lymphoma kinase

**Table 2:** Characteristics of advanced NSCLC patients treated with MWA plus chemotherapy

Patients’ characteristics	Overall (*n* = 148)	Training data set (*n* = 112)	External validation data set (*n* = 36)
Sex			
Male	96 (65%)	73 (65%)	23 (64%)
Female	52 (35%)	39 (35%)	15 (36%)
Age, years			
< 65	99 (67%)	72 (64%)	27 (75%)
≥ 65	49 (33%)	40 (36%)	9 (25%)
ECOG PS			
0–1	146 (99%)	110 (98%)	36 (100%)
2	2 (1%)	2 (92%)	0 (0%)
Smoking history			
Smokers	91 (61%)	69 (62%)	22 (61%)
Non-smokers	57 (39%)	43 (38%)	14 (39%)
Weight loss, ≥ 5% in the previous year			
Yes	13 (9%)	11 (10%)	2 (6%)
No	135 (91%)	101 (90%)	34 (94%)
Histology			
Adenocarcinoma	116 (78%)	92 (82%)	24 (67%)
Non-adenocarcinoma	32 (22%)	20 (18%)	12 (33%)
Clinical TNM stage			
IIIB	31 (21%)	23 (21%)	8 (22%)
IV	117 (79%)	89 (79%)	28 (78%)
Clinical T category			
0–2	97 (65%)	73 (65%)	24 (67%)
≥ 3	51 (35%)	39 (35%)	12 (33%)
Clinical N category			
0–1	39 (26%)	25 (22%)	14 (39%)
≥ 2	109 (74%)	87(78%)	22 (61%)
Tumor location			
Upper or middle lobe	80 (54%)	62 (55%)	18 (50%)
Lower lobe	68 (46%)	50 (45%)	18 (50%)
Tumor size, cm			
< 3.5	81 (55%)	66 (59%)	15 (42%)
≥ 3.5	67 (45%)	46 (41%)	21 (58%)
ALK status			
Wild type	14 (10%)	11 (10%)	3 (8%)
Mutant	5 (3%)	4 (4%)	1 (3%)
Unknown	128 (87%)	97 (84%)	32 (89%)
EGFR status			
Wild type	27 (18%)	19 (17%)	8 (22%)
Mutant	24 (16%)	18 (16%)	6 (17%)
Unknown	97 (66%)	75 (67%)	22 (61%)
Ablation power			
< 70 W	115 (78%)	86 (74%)	29 (81%)
70 W	33 (22%)	26 (26%)	7 (19%)
Ablation time, minute			
< 10	76 (51%)	56 (50%)	20 (56%)
≥ 10	72 (49%)	56 (50%)	16 (44%)
Ablation-related complications			
Yes	108 (73%)	94 (84%)	14 (39%)
No	40 (27%)	18 (16%)	22 (61%)
Chemotherapy regimen			
Pemetrexed	98 (66%)	82 (73%)	16 (44%)
Docetaxel	23 (16%)	19 (17%)	4 (11%)
Paclitaxel	7 (5%)	5 (5%)	2 (6%)
Gemcitabine	20 (13%)	6 (5%)	14 (39%)
Platinum category			
Cis-platinum	42 (28%)	12 (11%)	30 (83%)
Carboplatin	17 (12%)	11 (10%)	6 (17%)
Nedaplatin	89 (60%)	89 (79%)	0 (0%)

*ECOG PS*, Eastern Cooperation Oncology Group Performance Status. Ablation-related complications include fever, nausea, pneumothorax, pleural effusion, hemorrhage, infection, bronchopleural fistula, post-ablation syndrome, and nervous responses

### MWA procedure and chemotherapy

The MWA procedures were conducted by 15 chief physicians with  ≥ 5 years of experience in tumor ablation. To guide MWA, computed tomography (CT) was applied. MWA were operated with several common microwave ablation systems. The maximal output power we utilized was 70 W. Before the procedure, planning of the puncture point and the “target skin distance” for the target lesion was conducted. Once adequate anesthesia was achieved, an incision was made at the puncture point and the microwave antenna was inserted into the target lesion under CT guidance in accordance with the previous plan. If tumors were larger than 3.5 cm, two antennae would be utilized. Prior to initiating MWA, the cold circulating pipes, and pumps were connected to the antennae and machine. Technical success was determined by the attainment of post-ablation ground glass opacity measurements that exceeded the target lesion by a range of 5 to 10 mm. After 24–48 h of MWA, CT scans without enhancement were employed to screen whether there were ablation-related complications that required proper intervention.

The chemotherapy was generally conducted 7 days after the MWA procedure. Pemetrexed, paclitaxel, docetaxel, gemcitabine, or vinorelbine were administrated for chemotherapy. Cisplatin, nedaplatin, or carboplatin were applied as the corresponding platinum. The chemotherapy was performed by intravenous administration and repeated every 3 weeks and at most 6 cycles. Chemotherapy response was assessed every 6 weeks amid the therapy. Contrast-enhanced CT scans of the chest were conducted at 1-, 3-, 6-, 12-, 18-, 24-, and 36-month during the follow-up and repeated every 3 months after treatment was completed. All the detailed therapy-related product information can be found in eTable 1.

### Assessments

The key endpoint was progression-free survival (PFS) and the main secondary endpoint was overall survival (OS). In this study, PFS refers to the time from the beginning of therapy to disease progression or death, and OS refers to the time from the start of therapy to death. For patients who did not meet the endpoints, the censoring date was the date when their last clinical assessment was conducted. Other endpoints such as objective response rate (ORR) and time to local progression (TTLP) were recorded in the RCT but were excluded for the construction of this prediction model.

### Data processing

Continuous variables were transformed into categorical variables according to proper cutoff values. Age was cut at 65 years old to determine whether the patients belonged to the elderly. The cutoff value was set at 3.5 cm for tumor size as a tumor with a diameter larger than 3.5 cm has a high probability of failing to be completely ablated, thus two antennae were used. We chose 70 W and 10 min to cut off the ablation power and time respectively because ablation  ≥ 70 W or 10 min can lead to more ablation-related complications. Three missing values have been filled up by single imputation. If there were more than 10% of values were missing, this variable would be excluded from data analysis.

To build the model and conduct the validation, data collected from the hospitals located in Jinan City (Shandong Provincial Hospital; Shandong Academy of Medical Sciences; Jinan Military Region General Hospital) was made as the training data set, while the data from hospitals located in other cities was made as the external validation data set, which is completely distinct from the training data.

### Construction of the model

Statistical analysis was proceeded by R 4.2.1 for Windows (R Project for Statistical Computing; www.rproject.org). As lacking patients who reached the OS endpoint in the MWA plus chemotherapy group, PFS was eventually selected as the only endpoint. In the training data set, the LASSO regression process was used to conduct the multivariate analysis via the Cox regression model. To display the result, a forest plot of the selected variables was generated. The final variables were selected by step-down process, utilizing the Akaike information criterion as the stopping rule. After that, the nomogram was formulated by the survival and rms package in R.

### Validation and calibration of the model

The final model was subjected to 100 bootstraps resamples of the training data set and the external validation data set for internal and external validation respectively. The results of both internal and external validation were shown by the receiver operating characteristic (ROC) curve, concordance index (C-index), and Brier Score. The value of the C-index, which infers the area under the curve (AUC), ranges from 0.5 to 1.0. As 0.5 means a random chance and 1.0 means the model had a fully correct differentiating ability, it can be applied to assess the predicting ability of the final model. The value of the Brier Score ranges from 0 to 1.0, with higher values indicating better accuracy of the predicting model. To perform the calibration of the model for 1-year PFS, the predicted survival was compared with the observed survival.

### Risk group stratification

Risk group stratification was conducted using the score calculated by the nomogram to divide the entire data set into three risk groups. The high-, medium-, and low-risk groups included individuals whose scores were higher than the score of 30%, between the scores of 30 and 70%, and lower than the score of 70% 1-year PFS probability, respectively. Then the progression-free survival curves of each group were created by K-M estimates to compare the PFS. A simplified scoring system was built to provide a more convenient approach to evaluating the risks without decreasing the prediction accuracy of the original model. Moreover, a typical case was used to show the practicability of this system.

## Results

### Characteristics of patients

After selection, 148 patients (112 in the training data set and 36 in the validation data set) who were enrolled in the MWA and chemotherapy group in the RCT were included to build and validate the prediction model. There were 85 events (progression or deaths) over a median follow-up time of 13.1 months (95% confidence interval (CI), 10.2 to 16.5 months). The median progression-free survival time was 10.3 months (95% CI, 8.0 to 13.0 months). The detailed characteristics of included patients in the training and external validation data sets are presented in Table 2.

### Prognostic predictors of PFS in the training data set

**Fig. 1:**
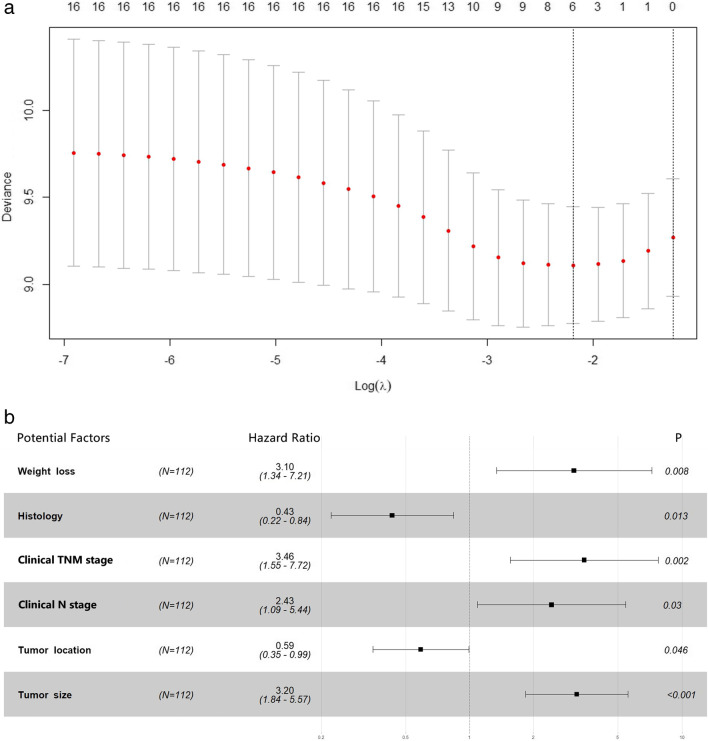
Results of LASSO regression. **a** Log(λ)-Deviance plot. The number of the selected variables is shown above the plot. When reaching the minimum deviance, the model built by the selected variables was the most optimal one. **b** Forest plot of the PFS (hazard ratio with 95% CI) of the selected variables

As there were more than 10% of values of EGFR and ALK status in the training data set were described as unknown, both of them were excluded from data analysis. The results of LASSO regression were displayed in Fig. 1. After multivariate analysis, we found six potential predictors, which were weight loss, histology, clinical TNM stage, clinical N category, tumor location, and tumor size.

### Development of the prognostic nomogram for PFS

**Fig. 2:**
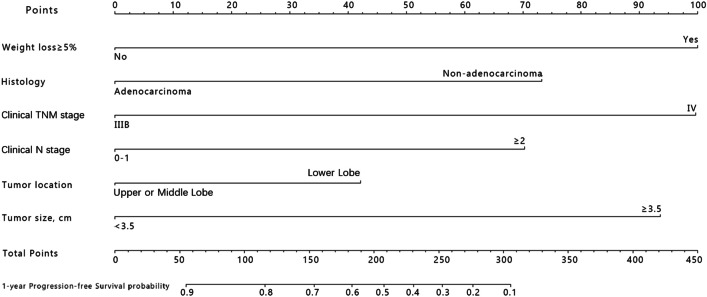
Nomogram for predicting PFS of advanced NSCLC patients treated with MWA plus chemotherapy. Usage: Above each variable axis locates a variable value. To calculate the score corresponding to each variable value, you need to draw a perpendicular line upward to read the number on the “Points” axis. The sum of the numbers needs to be found on the “Total Point” axis, then another perpendicular line needs to be drawn downward to acquire the 1-year progression-free survival probability

After the step-down process, a prognostic nomogram was built based on the selected predictors to determine the estimated probability of 1-year progress-free survival of patients who received MWA plus chemotherapy by calculating the total points (Fig. 2). From the points assigned to each factor, we found that weight loss, pathologic TNM stage, and tumor size were the primary ones contributing to prognosis, while the location was the least significant one.

### Validation and calibration of the prognostic nomogram

**Fig. 3:**
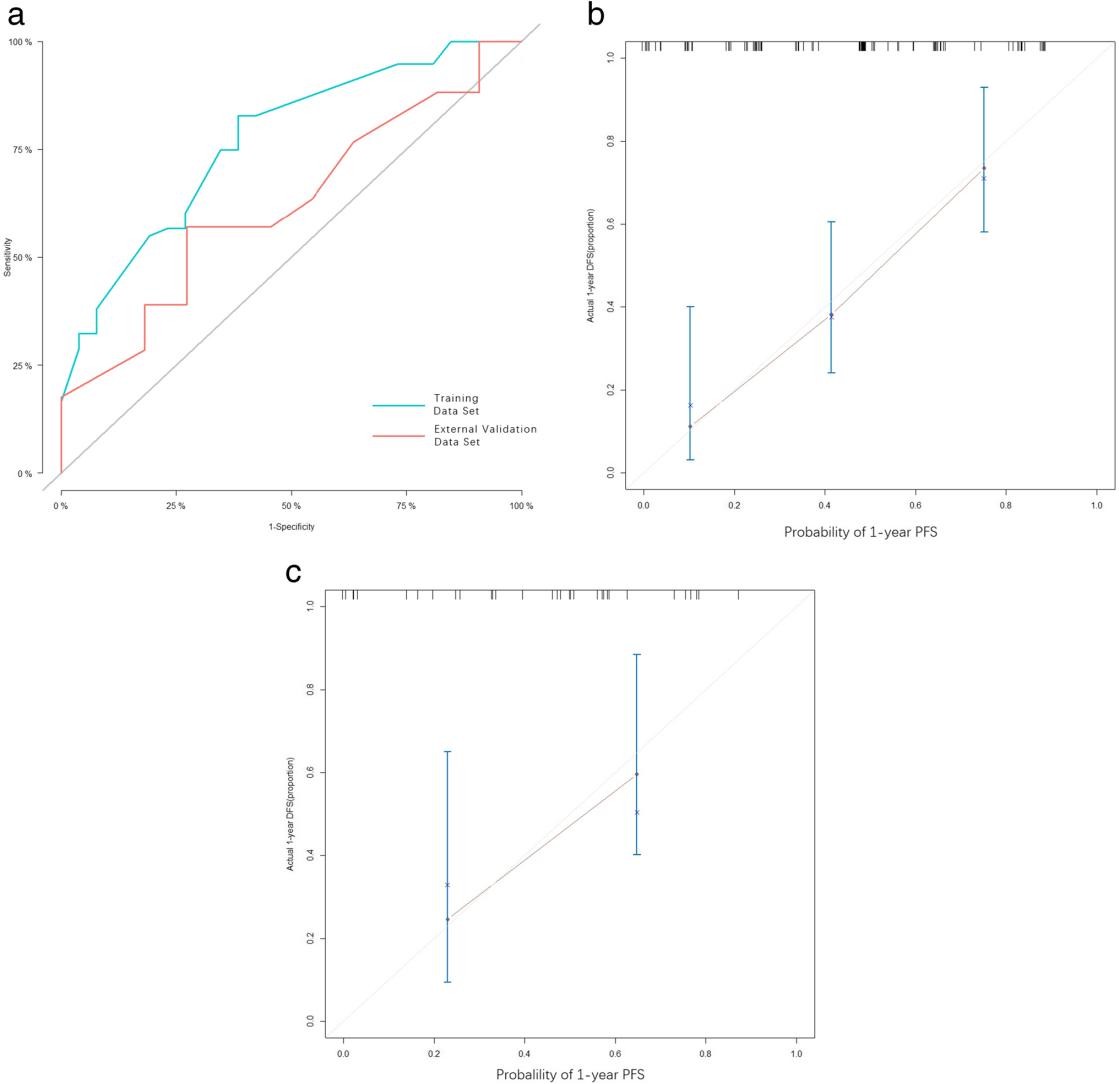
Statistical evaluation plots. **a** Receiver operating characteristic (ROC) curves of internal and external validation. The Sloping straight line demonstrated the reference value of AUC was 0.5. The C-index was calculated as the area between the ROC curve (blue or red curve) and the horizontal axis. The C-index was 0.77 (95% CI, 0.65–0.88) and 0.64 (95% CI, 0.43–0.85) in the training data set and external validation data set for predicting 1-year PFS, respectively. **b** Calibration curve of the training data set. The grey lines showed the reference values while the red lines are the curve-fitting lines. **c** Calibration curve of the external validation data set

In the internal validation, the C-index for this nomogram to predict PFS was 0.77 (95% CI, 0.65–0.88), while in the external validation, this index was 0.64 (95% CI, 0.43–0.85) (Fig. 3a). The Brier Scores of internal and external validation were 0.23 (95% CI, 0.14–0.33) and 0.25 (95% CI, 0.16–0.37), respectively. The calibration plots were shown in Fig. 3b, c.

### Risk-stratifying ability of the nomogram and the simplified scoring system

**Fig. 4:**
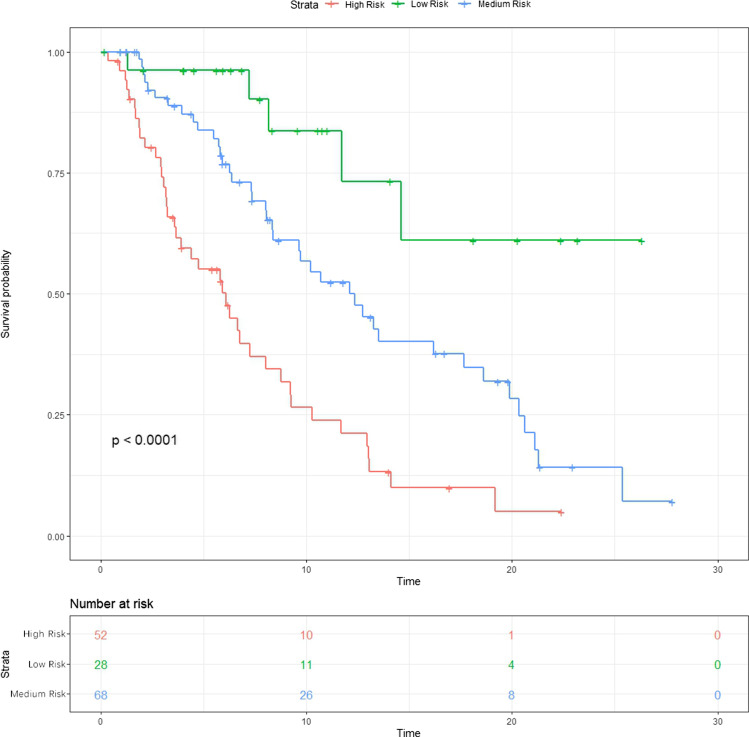
PFS of high-, medium-, and low-risk groups in the entire data set

**Fig. 5:**
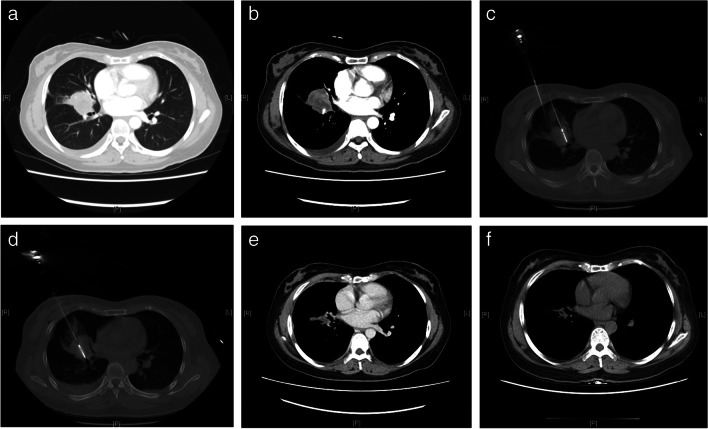
Imaging data of a typical case. **a** CT conducted before MWA. **b** Contrast-enhanced CT conducted before MWA. **c**, **d** CT during MWA. Two antennae were used. **e** Contrast-enhanced CT conducted 1 year after MWA. **f** Contrast-enhanced CT conducted 2 years after MWA

According to the nomogram, 152 and 252 corresponded to 70% and 30% 1-year PFS probability respectively and thus were selected as the cutting points to conduct risk group stratification. As shown in Fig. 4, the PFS of patients in the low-, medium-, and high-risk groups were considerably distinct (*p* < 0.0001). After simplifying the scores, we built a scoring system to make the group stratification more convenient (Table 3). The practicability of this system had been preliminarily validated through a typical case (Fig. 5). The pre-MWA CT and contrast-enhanced CT displayed that the tumor was located in the middle lobe and the maximum diameter of it was 3.9 cm. Two antennae were used to perform the MWA. The histopathological subtype was verified to be adenocarcinoma during the ablation. After MWA, the patient was treated with pemetrexed and nedaplatin. The total points calculated by the scoring system was 29, which indicated that the patient was stratified into the high-risk group and the estimated 1-year PFS probability was approximately 0.15. Although no local progression was detected by contrast-enhanced CT scans during the follow-up, pleural and brain metastases occurred and progressed, which led to the PFS of only 4.7 months.

**Table 3:** Simplified scoring system for evaluating PFS risks in advanced NSCLC patients treated with MWA plus chemotherapy

Patients’ characteristics	Points	Risk group
Weight loss, ≥ 5%		< 14
Yes	10	
No	0	Low-risk, 1-year PFS > 70%
Histology		
Adenocarcinoma	0	
Non-adenocarcinoma	7	
Clinical TNM stage		14~25
IIIB	0	
IV	10	Medium-risk, 1-year PFS 70%~30%
Clinical N category		
0–1	0	
≥ 2	7	
Tumor location		> 25
Upper or middle lobe	0	
Lower lobe	4	High-risk, 1-year PFS < 30%
Tumor size, cm		
< 3.5	0	
≥ 3.5	9	

Usage: Each variable value corresponds to a point. You need to add the points based on the patient’s characteristics and find the risk group according to the sum. Patients with points  < 14, 14~25, and  > 25 will be considered as low risk (1-year PFS > 70%), medium risk (1-year PFS 30%~70%), and low risk (1-year PFS < 30%), respectively

## Discussion

According to previous studies and clinical practice guidelines, combining thermal ablation and chemotherapy has manifested some gratifying advancements, such as improving local control rates of lung tumors and prolonging the survival of advanced NSCLC patients [5, 7–9]. However, the progression-free survival reported by previous studies ranged from 4.9 to 11.0 months, which showed a considerable difference [7, 12–15]. Thus, we sought to find the potential prognostic factors and build a prediction model to predict the PFS of advanced NSCLC patients treated with MWA and chemotherapy.

The data was obtained from a multi-center, randomized, controlled, phase III clinical trial. The centers involved are all tertiary A-level hospitals located in Shandong province, China, which provided excellent medical conditions to perform such trials. This guaranteed the data used to construct the prediction model was of good quality and representativeness.

From the model, we found that clinical TNM stage, tumor size, and weight loss are the three significant factors of PFS after MWA and chemotherapy in advanced NSCLC. According to The Eighth Edition IASLC Lung Cancer Stage Classification, the main difference between stage IIIB and stage IV is whether there is distant metastasis [16]. The median survival time of patients with stage IIIB and stage IV in the IASLC database is 10 months and 6 months, respectively [17]. Previous clinical trials showed that metastasis, as an independent prognostic factor, can lead to less PFS for advanced NSCLC patients, which was also supported by the results of our study [18, 19]. One possible reason to explain the relationship is that under most circumstances, metastatic lesions are chosen not to receive ablation when conducting MWA for advanced NSCLC patients. Thus, even though the primary lesions have been completely ablated, the metastatic lesions may progress and lessen the PFS. As for weight loss, two previous studies aiming to find the prognostic factors of advanced NSCLC have both considered it as a statistically significant factor that can affect PFS [20, 21]. Weight loss is considered to be a diagnostic criterion for cancer cachexia, which means that patients may suffer from systemic symptoms and have a lower tolerance for MWA and chemotherapy [22]. Tumor diameter has always been seen as an important prognostic risk factor for advanced NSCLC. For curative ablation, the maximum tumor diameter of proper candidates should be  ≤ 3 cm. However, most advanced NSCLC patients will receive palliative ablation, which allows patients with a tumor diameter of  > 3 cm to be treated with MWA [5]. Previous studies have found that tumor diameter mainly relates to local progression. One study performed in Japan showed that 32% of lung tumor lesions that were treated with thermal ablation developed local progression. In this study, a tumor diameter of  > 2 cm was considered as the predictor [23]. Another study conducted in China found that the local progression rate for advanced NSCLC patients who received MWA was 20.5%. Moreover, for the patients with tumor diameter  > 3 cm, the local progression rate surged to 81.3%, which was significantly higher than the general situation [24]. After univariable and multivariable analysis, we selected a tumor diameter of  ≥ 3.5 cm as the cutoff point because it could generate the highest C-index of the prediction model. It was also the criterion of applying two ablation antennae to perform the MWA in this trial. But whether using more than two antennae will affect the prognosis still needs further study.

In the model, clinical N stage, histology, and tumor location are the three predictors with relatively lower predictive efficiency. The Clinical N stage is an acknowledged risk factor for NSCLC. According to the IASLC database, a higher clinical N stage can lead to a worse prognosis [17]. To get the best predictive efficiency, we selected pathologic N stage  ≥ 2 as the cutoff point. As for histology, there is no clear evidence indicating that it is a risk factor that relates to the prognosis after conducting MWA. But previous chemotherapy studies have pronounced that patients with adenocarcinoma have more PFS benefits when compared to patients with non-adenocarcinoma [25, 26], which went some way towards explaining why histology was selected when constructing the prognostic prediction model of MWA plus chemotherapy. Interestingly, the results of our study demonstrated tumor location in the upper or middle lobe as a protective factor, which is in contradiction with the former model which aims to predict local progression after MWA in NSCLC patients [10]. As lacking relevant studies, the mechanisms by which the location of NSCLC can affect the prognosis of patients receiving MWA and chemotherapy still needs to be elucidated.

To build and validate the model, we divided the original data into two sets based on the center’s location where patients were engaged and treated in the RCT. It ensured the external validation data set conformed to the requirement of regional validation and improved the reliability of the validation. As the C-index of 0.7–0.8 and Brier Score of 0–0.25 is considered acceptable [27, 28], the validation indexes of the nomogram in the internal validation have fully met the expectation. Although the model showed less accuracy in the external validation data set, this circumstance is expected. After comparing the basic characteristics of data sets, we inferred the difference in sample size and follow-up time caused the discriminative ability to be reduced in external validation. The calibration curves proved there was no statistically significant differentiation between prediction and actual observation in both data sets. According to the PFS curves of high-, medium-, and low-risk groups, the risk-stratifying ability of the nomogram and scoring system was satisfying (*p* < 0.0001), which signified that both of them can be employed to anticipate the prognosis in clinical practice. We hope more model validation research, especially the ones that utilize data sets from other countries, can be done in the future to further validate the universality of this model.

To our knowledge, this is the first prediction model as well as the first published nomogram and scoring system for predicting the progression-free survival of advanced NSCLC patients who received MWA and chemotherapy. However, our study still has several limitations. First, due to the deficiency of original data, we failed to incorporate some potential prognostic factors (e.g., tumor cell differentiation, EGFR mutation, ALK-EML4 fusion) in variable analysis. Second, as the sample size of the external validation is relatively small, more data sets are warranted to better validate the reproductivity of the nomogram. Third, as the median follow-up time was 13.1 months, we failed to build a model that could predict 3- and 5-year PFS.

In conclusion, we found weight loss, histology, clinical TNM stage, clinical N category, tumor location, and tumor size were prognostic factors of progression after receiving MWA plus chemotherapy in advanced NSCLC patients. After building and validating, we published a nomogram that can predict 1-year PFS and a simplified scoring system for risk-group stratification. Hopefully, this model will assist physicians to predict the individualized PFS of their patients and decide whether to perform or terminate MWA and chemotherapy according to the expected benefits.

### Supplementary Information

Below is the link to the electronic supplementary material.

Supplementary file1 (PDF 28 KB)

## Abbreviations

C-index: Concordance index
CI: Confident interval
ECOG: Eastern Cooperation Oncology Group
EGFR: Epidermal growth factor receptor
EML4-ALK: Echinoderm microtubule–associated protein-like 4-anaplastic lymphoma kinase
K-M estimate: Kaplan-Meier estimate
MWA: Microwave ablation
NSCLC: Non-small cell lung cancer
ORR: Objective response rate
OS: Overall survival
PFS: Progression-free survival
PS: Performance status
ROC: Receiver operating characteristic
TTLP: Time to local progression

## Declarations

### Guarantor

The scientific guarantors of this publication are all three co-corresponding authors.

### Statistics and biometry

Fanhao Kong has significant statistical expertise.

### Study subjects or cohorts overlap

Some study subjects or cohorts have been previously reported in Wei Z, Yang X, Ye X et al (2020) Microwave ablation plus chemotherapy versus chemotherapy in advanced non-small cell lung cancer: a multicenter, randomized, controlled, phase III clinical trial. Eur Radiol 30:2692-2702.

### Methodology

• prospective

• randomized controlled trial

• multicenter study
