# Supplementary material for: A prognostic model for predicting progression-free survival in patients with advanced non-small cell lung cancer after image-guided microwave ablation plus chemotherapy
Source: Eur Radiol. 2023 Jun 15;33(11):7438–49. doi: 10.1007/s00330-023-09804-9 (PMC10598089; doi:10.1007/s00330-023-09804-9)
Supplement: Supplementary file 1 — Supplementary file1 (PDF 28 KB) [file 330_2023_9804_MOESM1_ESM.pdf]

A prognostic model for predicting progression-free survival in patients with advanced non–small cell lung cancer after image-guided microwave ablation plus chemotherapy

ELECTRONIC SUPPLEMENTARY MATERIAL

eTable 1. Information of devices and medications administrated for therapy

Devices and Medications	Company Information
Microwave Ablation Devices	
MTC-3C microwave ablation system	China Medical Devices R&D Center; Beijing, China
ECO-100A1 microwave ablation system	ECO Medical Instrument Co., Ltd; Nanjing, Jiangsu, China
KY-2450B microwave ablation system	CANYON Medical Inc.; Nanjing, Jiangsu, China
CT Devices	
Lightspeed 64 V	General Electric Co.; Boston, Massachusetts, USA
Medications	
Pemetrexed	Hansoh Pharma; Hong Kong SAR, China
Paclitaxel	Hengrui Medicine; Lianyungang, Jiangsu, China
Docetaxel	Qilu Pharma; Jinan, Shandong, China
Gemcitabine	Hansoh Pharma; Hong Kong SAR, China
Vinorelbine	Hansoh Pharma; Hong Kong SAR, China
Cisplatin	Qilu Pharma; Jinan, Shandong, China
Nedaplatin	Qilu Pharma; Jinan, Shandong, China
Carboplatin	Qilu Pharma; Jinan, Shandong, China
